# Association between atherosclerotic disease and cervical artery dissection in a population‐based cohort of older people

**DOI:** 10.1002/acn3.52216

**Published:** 2024-10-23

**Authors:** Joshua Kahan, Cenai Zhang, Ava L. Liberman, Alan Z. Segal, Santosh B. Murthy, Jiwon Kim, Hooman Kamel, Alexander E. Merkler

**Affiliations:** ^1^ Clinical and Translational Neuroscience Unit Feil Family Brain and Mind Research Institute and Department of Neurology, Weill Cornell Medicine New York New York USA; ^2^ Division of Cardiology Weill Cornell Medicine New York New York USA

## Abstract

**Objectives:**

Many cases of cervical artery dissection are considered “spontaneous.” Recent data suggest that while cervical artery dissection may proportionally explain more strokes in young patients, hospitalization for dissection increases with age, suggesting a potential role of acquired vascular disease. In this study, we hypothesized that traditional vascular risk factors and comorbidities are associated with cervical artery dissection.

**Methods:**

We performed a retrospective cohort study using administrative claims data from a 5% sample of Medicare beneficiaries. Exposures of interest included traditional vascular risk factors and comorbidities: coronary artery disease, hyperlipidemia, hypertension, diabetes mellitus, heart failure, chronic kidney disease, chronic obstructive pulmonary disease, valvular heart disease, atrial fibrillation, tobacco use, and alcohol abuse. The primary outcome was a new diagnosis of cervical artery dissection. Marginal structural Cox models were used to characterize the association between the exposures and outcomes, adjusted for time‐dependent confounding.

**Results:**

Among 2,256,710 eligible Medicare beneficiaries, 730 (0.03%) developed cervical artery dissection. The following exposures were found to be significantly associated with the development of cervical artery dissection: hypertension (HR 1.84 [95% CI: 1.40–2.41]), alcohol use (HR 1.83 [1.52–2.21]), atrial fibrillation (HR 1.80 [1.53–2.11]), tobacco use (HR 1.80 [1.52–2.13]), coronary artery disease (HR 1.56 [1.33–1.82]), and valvular heart disease (HR 1.23 [1.05–1.45]).

**Interpretation:**

In a large cohort of older people, several traditional vascular risk factors and comorbidities were associated with subsequent cervical artery dissection. Further studies exploring the role of such factors in the development of cervical artery dissection are warranted.

## Introduction

Cervical artery dissection represents an important cause of ischemic stroke in younger people. Observational studies in North America estimate the annual incidence rate to be roughly 2.6–3.0 per 100,000 population,^1^ with carotid dissection being almost twice as common as vertebral dissection. As many dissections produce little or no symptoms, the true incidence is likely greater.^2^, ^3^ Although dissection may explain a larger proportion of strokes in younger patients, hospitalization for dissection‐related stroke increases with age.^4^ One possible reason for this could be that acquired atherosclerotic vascular disease may play a role in dissection.

Established risk factors for cervical artery dissection include trauma, both major^5^ and minor,^6^, ^7^, ^8^, ^9^, ^10^ recent infection,^11^, ^12^, ^13^, ^14^ vascular morphological features of the carotid artery such as evidence of redundancies (kinks and loops),^6^, ^15^, ^16^ or the presence of connective tissue diseases (e.g., Ehlers–Danlos syndrome, Marfan syndrome, or osteogenesis imperfecta).^7^, ^17^, ^18^, ^19^ However, the role of traditional vascular risk factors and comorbidities which typically accumulate with age is uncertain. Case–control studies addressing these questions have mostly been limited to young cohorts, in whom hypertension has been shown to be associated with cervical artery dissection.^20^, ^21^ Prospective data from young people with cervical dissections has similarly shown hypertension increases the risk of dissection,^22^ and Mendelian randomization approaches have suggested possible causal relationships between hypertension and cervical artery dissection.^23^ The role of other vascular risk factors remains unclear, especially in older people.

Given that the prevalence of hospitalization for dissection‐related strokes increases across the lifespan,^4^ developing a firmer understanding of its risk factors could provide insight into underlying pathophysiological mechanisms and new targets for prevention and treatment. We therefore sought to evaluate the association of traditional vascular risk factors and comorbidities with cervical artery dissection in a large, population‐based cohort of older adults.

## Methods

### Design

We performed a retrospective cohort study using inpatient and outpatient administrative claims data from a 5% sample of Medicare beneficiaries from 1 January 2008 through 31 December 2018. The Centers for Medicare and Medicaid Services (CMS) provide health insurance to roughly 70% of US residents 65 years of age or older. Healthcare provider claims for individual patient services are submitted to CMS and then organized into deidentified datasets for research use; unique patient identifiers allow for longitudinal linkage across different healthcare settings and facilities. Individual claims include dates of service and International Classification of Diseases, 9th Revision or 10th Revision, Clinical Modification (ICD‐9‐CM or ICD‐10‐CM) diagnosis codes. Weill Cornell Medicine's institutional review board approved this analysis of deidentified data and waived the requirement for informed consent. The data used in this analysis are restricted per the terms of the CMS data use agreement. Investigators can obtain access to these data by application to the CMS.

### Population

We included patients with continuous Medicare coverage (Parts A and B) for at least 1 year or until death.

### Measurements

The exposures of interest were traditional vascular risk factors and comorbidities: hypertension, hyperlipidemia, coronary heart disease, diabetes mellitus, heart failure, peripheral vascular disease, chronic kidney disease, chronic obstructive pulmonary disease, valvular heart disease, atrial fibrillation, obstructive sleep apnea, tobacco use, and alcohol abuse. These risk factors were identified through ICD‐9‐CM or ICD‐10‐CM diagnostic code algorithms used in prior work,^24^, ^25^, ^26^ which have previously been validated against medical record review with >95% positive predicted values.^27^ Both inpatient and outpatient claims were searched for diagnostic codes as many of these exposures are chronic conditions primarily managed on an outpatient basis. History of stroke, identified using the same method,^28^ was also included as a potential confounding exposure.

The primary outcome was a new diagnosis of cervical artery dissection, as identified by inpatient claims with ICD‐9‐CM codes 443.21 and 443.24, or ICD‐10‐CM codes 177.71 and 177.74 in any discharge diagnosis position. The ICD‐9‐CM codes have previously been validated to have a positive PPV of 82.1% for dissection,^29^ with sensitivities estimated to be 80–85% and specificity approaching 100%.^4^

### Statistical analysis

We used survival analysis and patients were followed until the time of death, end of Medicare coverage, or date of last available data (December 31, 2018). The conventional method for estimating the effect of a time‐varying exposure is to use a time‐dependent Cox proportional hazards model, which models the hazard of failure as a function of past exposure history. However, research suggests that this approach may be biased, regardless of whether past covariate history is adjusted for, if (1) a time‐dependent covariate exists that is both a risk factor for the outcome and predicts subsequent exposure, and (2) past exposure history predicts the risk factor.^30^, ^31^ In our study, some of the putative risk factors were common diseases that could complicate the relationship between other risk factors and cervical artery dissection by interacting with or confounding the association.^32^ Marginal structural models are a type of causal model that utilizes inverse probability weighting to estimate unbiased parameters for time‐varying exposures in observational data while accounting for time‐dependent confounding variables.^33^ To provide more robust risk estimates, we decided to use the marginal structural Cox proportional hazards model with stabilized inverse probability weight in all analyses. Robust (sandwich) standard errors were used for computing confidence intervals. The marginal structural models included all the exposures above along with the covariates of prior stroke, age, sex, and race/ethnicity.

We limited our cohort to individuals who maintained continuous coverage in traditional fee‐for‐service Medicare (Parts A and B) for a minimum of 1 year or until death, aligning with standard practice of analyzing Medicare data. While Medicare eligibility commences at 65 years of age, our study encompassed only patients aged 66 or older. This criterion allowed sufficient time for beneficiaries to engage in medical care, and for healthcare providers to record any pre‐existing medical comorbidities, self‐reported or otherwise, including a prior cervical artery dissection. We captured vascular risk factors and comorbidities at baseline (i.e., when subjects first enrolled in Medicare) and included these baseline variables in our marginal structural model. In our model, each risk factor has two variables: one is fixed, representing the baseline value, and the other is time‐varying, representing the changing comorbidity over time. In other words, we adjust for risk factors based on both baseline values and time‐dependent changes.

We performed three sensitivity analyses to test the robustness of associations. First, we used both inpatient and outpatient claims instead of inpatient claims only to define the diagnosis of cervical artery dissection. Second, we excluded patients who had trauma when the new diagnosis cervical artery dissection was present. Third, we excluded patients who had a history of stroke prior to onset of their dissection diagnosis (i.e., strokes prior to Medicare enrollment, and strokes occurring during the follow‐up period but prior to the diagnosis of dissection.) All the analyses were performed using R, version 4.2.3 (R Foundation for Statistical Computing, Vienna, Austria) and Stata/MP, version 15.1 (Stata Corp, TX). For the primary analyses, *p* values were corrected for multiple comparisons testing using the False Discovery Rate (FDR) correction.

## Results

### Patient characteristics

We included 2,256,710 Medicare beneficiaries with a mean age of 71.5 years, of whom 56.3% were women (Table 1). Of the total cohort, 730 patients (0.03%) received a diagnosis of cervical artery dissection. The mean age for patients with a cervical artery dissection diagnosis was 71.2 years (standard deviation [SD] = 7.0), compared to a mean age of 71.5 years (SD 8.0) for those without a cervical artery dissection diagnosis. The demographics and prevalence of atherosclerosis risk factors in both groups at the time of enrollment in Medicare are summarized in Table 1. As would be expected, the most prevalent risk factors in both groups were hypertension, hyperlipidemia, diabetes, and coronary artery disease.

**Table 1:** Baseline demographics and prevalence of vascular risk factors.

	No dissection	Dissection
	*N* = 2,255,980	*N* = 730
Age, mean (SD), years	71.5 (8.0)	71.2 (7.0)
Female (%)	1,270,682 (56.3)	317 (43.4)
Race		
White	1,914,507 (84.9)	634 (86.9)
Black	185,903 (8.2)	64 (8.8)
Other	155,570 (6.9)	32 (4.4)
Atrial fibrillation/flutter	151,489 (6.7)	61 (8.4)
Coronary heart disease	340,225 (15.1)	150 (20.6)
Hypertension	1,051,046 (46.6)	379 (51.9)
Diabetes mellitus	448,928 (19.9)	162 (22.2)
Congestive heart failure	133,673 (5.9)	46 (6.3)
Peripheral vascular disease	138,107 (6.1)	73 (10.0)
Chronic kidney disease	98,391 (4.4)	36 (4.9)
Chronic obstructive pulmonary disease	217,253 (9.6)	59 (8.1)
Valvular heart disease	130,568 (5.8)	54 (7.4)
Tobacco use	41,099 (1.8)	21 (2.9)
Alcohol abuse	62,587 (2.8)	38 (5.2)
Hyperlipidemia	828,923 (36.7)	300 (41.1)
Prior stroke	51,651 (2.3)	36 (4.9)

A retrospective cohort using inpatient and outpatient administrative claims data from a 5% sample of Medicare Beneficiaries, stratified by diagnosis of cervical artery dissection (primary analysis). The vascular risk factors listed were extracted from inpatient and outpatient claims at the time of enrollment in Medicare. The mean age of the cohort is 71.5 years. Continuous data is presented as mean (standard deviation [SD]). Discrete data are presented as count (percentage).

### Primary analysis

In our marginal structural Cox model, the following exposures were associated with a subsequent diagnosis of cervical artery dissection (Table 2): hypertension (HR 1.84 [95% CI: 1.40–2.41]), alcohol use (HR 1.83 [1.52–2.21]), atrial fibrillation (HR 1.80 [1.53–2.11]), tobacco use (HR 1.80 [1.52–2.13]), coronary artery disease (HR 1.56 [1.33–1.82]), and valvular heart disease (HR 1.23 [1.05–1.45]).

**Table 2:** Hazard ratios of comorbidities as they relate to subsequent diagnosis of cervical artery dissection.

	Primary analysis			
	*N* dissections = 730			
	HR	95% CI	*p* value	FDR *p* value
Hypertension	1.84	1.40–2.41	<0.001	<0.001
Alcohol abuse	1.83	1.52–2.21	<0.001	<0.001
Atrial fibrillation	1.80	1.53–2.11	<0.001	<0.001
Tobacco use	1.80	1.52–2.13	<0.001	<0.001
Coronary artery disease	1.56	1.33–1.82	<0.001	<0.001
Valvular heart disease	1.23	1.05–1.45	0.012	0.021
Congestive heart failure	1.22	1.02–1.46	0.034	0.054
Chronic kidney disease	1.19	0.99–1.41	0.058	0.079
Hyperlipidemia	1.12	0.91–1.38	0.292	0.357
Diabetes	1.06	0.91–1.23	0.487	0.536
Chronic obstructive pulmonary disease	0.96	0.82–1.12	0.606	0.606

Hazard ratios (HR) and corresponding 95% confidence intervals (CI), *p* values, and False Discovery Rate (FDR) corrected *p* values for the primary analysis. The primary analysis defined the outcome (cervical artery dissection) using inpatient claims only.

### Sensitivity analyses

**Figure 1:**
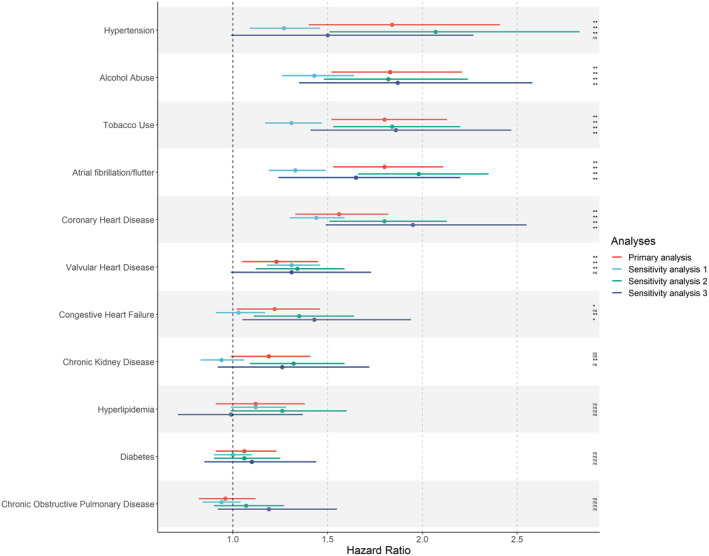
Hazard ratios and corresponding confidence intervals (CI) for the exposures explored, from all three analyses. The primary analysis (red) defined the outcome (cervical artery dissection) using inpatient claims only. Sensitivity analysis 1 (blue) used both inpatient and outpatient claims to define the outcome. Sensitivity analysis 2 (green) defined the outcome as per the primary analysis but excluded patients who had trauma when the new diagnosis of cervical artery dissection was present. Sensitivity analysis 3 (navy) excluded all patients with prior stroke from the analysis. *p < 0.05. **FDR *p* < 0.05. ns, not statistically significant.

The associations between atherosclerotic risk factors and incident cervical artery dissection were minimally changed when we altered the way we defined cervical artery dissection, specifically by using both inpatient and outpatient claims to define our primary outcome compared to simply using inpatient claims (Fig. 1). Table 3 lists respective hazard estimates of all three analyses. Hazard estimates were generally attenuated after the inclusion of outpatient claims, however, the general direction and relative magnitudes were remained similar (Sensitivity analysis 1, Table 3).

**Table 3:** Sensitivity analyses.

	Sensitivity analysis 1				Sensitivity analysis 2				Sensitivity analysis 3			
	Dissection *N* = 1715				Dissection *N* = 594				Dissection *N* = 251			
	HR	95% CI	*p* value	FDR *p* value	HR	95% CI	*p* value	FDR *p* value	HR	95% CI	*p* value	FDR *p* value
Hypertension	1.27	1.09–1.46	0.002	0.003	2.07	1.51–2.83	<0.001	<0.001	1.5	0.99–2.27	0.055	0.087
Alcohol abuse	1.43	1.26–1.64	<0.001	<0.001	1.82	1.48–2.24	<0.001	<0.001	1.87	1.35–2.58	<0.001	<0.001
Atrial fibrillation	1.33	1.19–1.49	<0.001	<0.001	1.98	1.66–2.35	<0.001	<0.001	1.65	1.24–2.20	<0.001	<0.001
Tobacco use	1.31	1.17–1.47	<0.001	<0.001	1.84	1.53–2.20	<0.001	<0.001	1.86	1.41–2.47	<0.001	<0.001
Coronary artery disease	1.44	1.30–1.59	<0.001	<0.001	1.80	1.51–2.13	<0.001	<0.001	1.95	1.49–2.55	<0.001	<0.001
Valvular heart disease	1.31	1.18–1.46	<0.001	<0.001	1.34	1.12–1.59	0.001	0.002	1.31	0.99–1.73	0.055	0.087
Congestive heart failure	1.03	0.91–1.17	0.646	0.711	1.35	1.11–1.64	0.002	0.003	1.43	1.05–1.94	0.025	0.055
Chronic kidney disease	0.94	0.83–1.06	0.317	0.387	1.32	1.09–1.59	0.003	0.004	1.26	0.92–1.72	0.154	0.212
Hyperlipidemia	1.12	0.99–1.28	0.080	0.126	1.26	0.99–1.60	0.051	0.062	0.99	0.71–1.37	0.942	0.942
Diabetes	1.00	0.90–1.10	0.925	0.925	1.06	0.90–1.25	0.347	0.382	1.1	0.85–1.44	0.461	0.507
Chronic obstructive pulmonary disease	0.94	0.84–1.04	0.202	0.278	1.07	0.90–1.27	0.393	0.393	1.19	0.92–1.55	0.191	0.233

Hazard ratios (HR) and corresponding 95% confidence intervals (CI) for three analyses. The primary analysis defined the outcome (cervical artery dissection) using inpatient claims only. Sensitivity analysis 1 used both inpatient and outpatient claims to define the outcome. Sensitivity analysis 2 defined the outcome as per the primary analysis, but excluded patients who had trauma when the new diagnosis cervical artery dissection was present. Sensitivity analysis 3 excluded all patients with prior stroke from the analysis.

After excluding the patients who experienced trauma concurrent with cervical artery dissection, the results remained similar (Sensitivity analysis 2, Table 3). After excluding patients with a prior stroke, results were again qualitatively similar, although certain variables including hypertension and valvular heart disease marginally no longer met statistical significance (Fig. 1). Notably, after excluding patients with prior stroke, the number of dissections captured dropped from 730 to 251 (Sensitivity analysis 3, Table 3).

## Discussion

Using a large population‐based cohort of older people across the United States, we found that patients with traditional vascular risk factors and comorbidities were more likely to develop cervical artery dissection.

Prior studies evaluating the association between traditional vascular risk factors and dissection have yielded mixed results. The CADISP consortium study compared the prevalence of vascular risk factors in European patients with cervical artery dissection, patients with non‐dissection‐related ischemic strokes, and healthy controls. They found that patients with dissection tended to have higher rates of hypertension and lower rates of dyslipidemia and obesity.^21^ There was a tendency for dissection patients to be more likely to smoke; however, this association did not reach statistical significance. In the same cohort, the frequency of vascular risk factors increased with age in both dissection and non‐dissection patients with ischemic stroke, but more so in the non‐dissection stroke cohort.^13^ In another European case–control study, patients with dissection tended to have more hypertension, although this association did not reach statistical significance.^20^ However, there are other studies that have found the opposite.^10^, ^34^ A recent prospective trial in young patients (mean age 47 years) demonstrated that hypertension and migraine were associated with an increased risk of cervical artery dissection, whereas diabetes and hyperlipidemia were associated with a decreased risk of dissection.^22^

In this context, our study provides novel findings on associations between a broad range of traditional vascular risk factors and the risk of cervical artery dissection in older adults (mean age 71 years). Given the paucity of previous data on the role of vascular disease in cervical dissection in older patients, we employed a population‐level approach using causal modeling, and our findings implicate several risk factors that have not previously been evidenced. As seen in prior studies,^21^ our work identified hypertension as an important risk factor for the development of cervical artery dissection. Additionally, our results highlight risks posed by tobacco use, alcohol use, coronary and valvular heart disease, as well as atrial fibrillation in the development of cervical artery dissection. The role of tobacco use in carotid artery atherosclerosis is well established^35^; however, its relationship to dissection has not previously been seen. The relationship between alcohol use and cervical artery dissection has also not previously been demonstrated and appears to be independent of recorded trauma on the basis of our sensitivity analysis. The relationship between valvular heart disease and dissection is also notable. Prior work has identified that aortic root diameter enlargement is associated with an increased risk of spontaneous cervical artery dissection, consistent with the idea that there could be a generalized defect in the vascular extracellular matrix.^36^, ^37^

The observation that hypertension no longer met statistical significance when prior stroke was excluded and that the number of dissections captured dropped significantly, suggests that the relationship between hypertension and cervical artery dissection may be partly mediated or confounded by the presence of a prior stroke. This finding emphasizes the complex interplay between traditional vascular risk factors and the occurrence of dissections, particularly in older adults. It remains to be seen whether this finding is due to the loss of statistical power in this sensitivity analysis, or whether there is a true interaction between prior stroke and hypertension. However, given the consistent qualitative trends in all three analyses, it is difficult to ignore the potential risk of hypertension, regardless of a prior stroke diagnosis.

Our findings have several potential implications. First, from a mechanistic perspective, our data suggest that traditional vascular risk factors, perhaps partly by accelerating the process of atherosclerosis, may make the carotid and vertebral arteries vulnerable to intimal tears and intramural hemorrhage. This mechanism has been widely accepted in acute aortic dissection^37^, ^38^ and has been cited in some cases of spontaneous coronary artery dissection^39^, ^40^ but has not been previously hypothesized for cervical artery dissection. As in the aorta, our results suggest that atherosclerosis may cause the same vulnerability in the cervical arteries.

Second, from a practical standpoint, many of the previously established risk factors for cervical artery dissection, such as unspecified collagen vascular disease, are currently largely unmodifiable.^41^ In contrast, our results suggest several risk factors for dissection that can be modified with a combination of lifestyle choices and medical management. Given the absolute incidence of cervical artery dissection has been shown to increase with age^4^; it is important to reinforce that prevention of atherosclerotic diseases might reduce future risk of cervical artery dissection.

Third, if confirmed, our results may lead to changes in the etiological classification of cervical artery dissection and their resultant strokes per TOAST criteria.^42^ In addition to trauma and connective tissue disorders, traditional acquired vascular disease may also be a causative etiology of cervical artery dissection. This may lead to changes in the management of cervical artery dissection beyond simply antithrombotic therapy. Given our previous findings that cervical artery dissection is associated with a heightened risk of aortic dissection,^37^ the data may indicate that patients with cervical artery dissection may benefit from therapies to reduce atherosclerosis (lipid‐lowering medications, antihypertensive drugs) as well as evaluation of systemic atherosclerotic disease to mitigate the risk of future major vascular events.

### Study limitations

Our study should be considered in light of its limitations. The use of Medicare claims data means our findings are vulnerable to misclassification errors. To mitigate the risk of misclassification, we used validated diagnostic codes to identify dissection outcomes. Similarly, while this study estimated the risk associated with certain factors, we are unable to truly assess the underlying pathology of cervical artery dissection. For example, while alcohol use appears to confer a significant risk to the development of cervical artery dissection, it is conceivable that it might also cause people to fall over and sustain head and neck trauma,^43^ which might be more important to the development of cervical artery dissection than any inherent vasculopathy alcohol use may produce. Despite this, our results were similar in a sensitivity analysis in which we excluded patients with trauma. Another consideration is that the ICD codes defining our outcome are not specific to extracranial carotid of vertebral dissections, and so in theory a subset of these patients could have had intracranial dissections. Further, we were unable to determine if dissection involved the vertebral or carotid artery given our exclusive reliance on administrative data. Our methods assume that the medical diagnoses collected at baseline were complete and accurate, and any subsequently added diagnoses were considered newly acquired. If this assumption were invalid, then the patients included may present different vascular risk factor profiles, potentially influenced by a previous unmeasured dissection. It would also mean that some of the outcome events represent a recurrence of a dissection instead of a new dissection, which may be a mechanistically independent process. Finally, it is important to highlight that the risk factors identified herein were in an older cohort of patients, and so these data might not generalize to a younger population.

In a large population‐based cohort of older adults, we found that traditional vascular risk factors and comorbidities were associated with the development of cervical artery dissection. Our study suggests that cervical artery dissection, particularly in older people, may result from an arteriopathy caused by acquired vascular injury from hypertension‐ and atherosclerosis‐related pathology. Our results may imply that there are modifiable risk factors for cervical artery dissection in older people that could be the focus of future efforts to prevent and treat cervical artery dissection.

## Author Contributions

JKahan, CZ, HK, and AEM contributed to conception and design of the study. CZ, HK, and AEM contributed to acquisition and analysis of data. JKahan, CZ, ALL, AZS, SBM, JKim, HK, and AEM contributed to drafting a significant portion of the manuscript or figures.

## Conflict of Interest

Disclosures: J Kahan, CZ, and J Kim declare that they have no competing interests. AZS, SM, and AEM have served as expert consultants on neurological disorders. ALL is supported by the NIH/NINDS (grant K23NS107643) and the Victor and Tara Menezes Clinical Scholar in Neuroscience award. SM is in receipt of NIH funding (K23NS105948). HK serves as a PI for the NIH‐funded ARCADIA trial (NINDS U01NS095869), which receives in‐kind study drugs from the BMS‐Pfizer Alliance, and ancillary study support from Roche Diagnostics; on clinical trial steering/executive committees for Medtronic, Janssen, and Javelin Medical; and on endpoint adjudication committees for AstraZeneca, Novo Nordisk, and Boehringer Ingelheim. HK has an ownership interest in TETMedical, Inc.

## Data Availability

The data used for this analysis cannot be directly shared by the authors under the terms of their data use agreement, but the data can be obtained by application to the Centers for Medicare & Medicaid Services.
